# Comparison of classification algorithms for predicting autistic spectrum disorder using WEKA modeler

**DOI:** 10.1186/s12911-022-02050-x

**Published:** 2022-11-24

**Authors:** Siti Fairuz Mohd Radzi, Mohd Sayuti Hassan, Muhammad Abdul Hadi Mohd Radzi

**Affiliations:** 1 Centre for Global Sustainability Studies, Universiti Sains Malaysia, Minden, Malaysia; 2 School of Languages, Literacies, and Translation, Universiti Sains Malaysia, Minden, Malaysia

**Keywords:** Data mining, Healthcare, Autistic spectrum disorder, Classification, Missing values

## Abstract

**Background:**

In healthcare area, big data, if integrated with machine learning, enables health practitioners to predict the result of a disorder or disease more accurately. In Autistic Spectrum Disorder (ASD), it is important to screen the patients to enable them to undergo proper treatments as early as possible. However, difficulties may arise in predicting ASD occurrences accurately, mainly caused by human errors. Data mining, if embedded into health screening practice, can help to overcome the difficulties. This study attempts to evaluate the performance of six best classifiers, taken from existing works, at analysing ASD screening training dataset.

**Result:**

We tested Naive Bayes, Logistic Regression, KNN, J48, Random Forest, SVM, and Deep Neural Network algorithms to ASD screening dataset and compared the classifiers’ based on significant parameters; sensitivity, specificity, accuracy, receiver operating characteristic, area under the curve, and runtime, in predicting ASD occurrences. We also found that most of previous studies focused on classifying health-related dataset while ignoring the missing values which may contribute to significant impacts to the classification result which in turn may impact the life of the patients. Thus, we addressed the missing values by implementing imputation method where they are replaced with the mean of the available records found in the dataset.

**Conclusion:**

We found that J48 produced promising results as compared to other classifiers when tested in both circumstances, with and without missing values. Our findings also suggested that SVM does not necessarily perform well for small and simple datasets. The outcome is hoped to assist health practitioners in making accurate diagnosis of ASD occurrences in patients.

## Introduction

Autistic Spectrum Disorder (ASD), which was first described in 1940, is a group of neuro-developmental disorders that commonly occurs in boys [1]. Children with ASD will commonly develop a number of peculiar behavioural changes and will have difficulties in social interaction and communication which will become more apparent when the children reach six months of age [2–4]. There is a need to ensure that ASD is diagnosed at the earliest stage in order to facilitate and prepare the parents or caregivers of the affected children with appropriate interventions as soon as possible [4]. Early interventions for patients diagnosed with ASD are important so as to ensure that the patients and caregivers are able to deal with the environmental circumstances during the growing-up phase of the children. Thus, prompt classification of ASD in children is very important which makes the integration of screening tool with data mining tool crucial. A systematic review conducted by Marlow et al. [5] showed that the screening tools that have been used by healthcare practitioners have yet to incorporate machine learning intervention intensively. According to Pinto-Martin et al. [6], there is a need to ensure that a tool is incorporated in the development of ASD screening tool for rapid and accurate diagnosis since medical practitioners may probably have difficulties to keep up with the screening process all by themselves which can lead to human errors and in this case, machine learning method can help to solve the issue [7].

There is an urgent need to ensure that machine learning is incorporated in healthcare area to assist the practitioners, however, not many studies have been conducted to examine the sensitivity, specificity, and accuracy of machine learning techniques for ASD screening process which makes the integration nearly impossible. This has been mentioned by Thabtah [8] who compiled a number of ASD screening-related datasets which can be analysed through machine learning for improving the sensitivity, specificity, and accuracy in diagnosing ASD patients. In other similar research, Thabtah [9] found that the algorithms in machine learning can assist practitioners in expediting the process of screening and diagnosing a patient with ASD. Thus, we attempt to fill in the gap by conducting an experiment to compare the best classification algorithms to be used for ASD screening process.

This paper is organized as follows; "Research Method" section explains the approaches that we used to classify the ASD screening dataset including the classification process, classification techniques, performance matrix, and dataset involved in this study, "Result and discussion" section presents the results of the classification process using WEKA Explorer and WEKA Experimenter to the dataset in two conditions; with and without missing values, "Conclusion" section concludes the findings and suggests several potential studies that can be conducted in the future.

### Motivation

Not many studies have utilized classification algorithms to particularly analyse ASD screening dataset. From the literature review, most researchers utilized other most common datasets such as breast cancer and heart disease datasets, thus there exists difficulties in analysing and comparing the performance of classifiers being tested to ASD screening dataset. This is important in order to assist in selecting the best classification methods for screening and diagnosing a patient with ASD, thus we attempted to fill in the gap by analysing and classifying ASD screening dataset by using six classifiers.

### Related works

There have been a number of studies conducted to investigate the role of data mining or data science in assisting healthcare practitioners in analysing and diagnosing a particular disease or illness in an effective and efficient manner. Many countries have been utilizing health informatics as a way of reducing the government spending on healthcare data management. In the United States, health informatics has helped the government to save the medical management industry up to $450 billion each year annually [10]. This is due to the fact that health informatics is capable of integrating big healthcare data from different sources which is useful to assist the healthcare practitioners particularly in detecting or diagnosing a disease or illness more accurately.

The utilization of machine learning for data mining can assist professionals to examine medical or health related problem that can lead to better understanding of healthcare related issues [11]. In data mining, several methods are utilized to analyse healthcare dataset especially for classifying a particular disease; statistical analysis, decision tree, k-nearest neighbour, artificial neural network, and many more [12].

There is an urgent need to ensure that machine learning is incorporated in assisting the practitioners, however, little studies were conducted to examine the sensitivity, specificity, and accuracy of machine learning techniques for ASD screening process which makes the integration nearly impossible. This has been mentioned by Thabtah [8] who compiled a number of ASD screening-related datasets which can be analysed through machine learning for improving the sensitivity, specificity, and accuracy in diagnosing ASD patients. This study attempted to fill in the gap by conducting the experiment in comparing the best classification algorithms to be used for ASD screening. In other similar research, Thabtah [13] found that the algorithms in machine learning can assist practitioners in expediting the process of screening and diagnosing a patient with ASD.

Ramotra et al. [14] conducted a study to predict the presence of heart disease by utilizing classification techniques namely Decision Tree, Naïve Bayes, Support Vector Machines, and Artificial Neural Networks classifiers. The study concluded that Naive Bayes classifier presented the highest accuracy among other classifiers being tested. A study conducted by Kibis et al. [15] utilized artificial neural network (ANN), classification and regression tree (C&RT), Logistic Regression, and Bayesian Belief Network (BBN) to investigate breast cancer survivability with respect to the classifiers’ accuracy, sensitivity, specificity, and area under the curve (AUC) metric. The study found that Logistic Regression method presented better performance with regard to AUC metrics. Alaiad et al. [16] tested five classification algorithms, namely, Naive Bayes, Decision Tree, Support Vector Machine, K-Nearest Neighbour (KNN), and JRip in predicting chronic kidney disease. The study found that KNN achieved high performance in term of the accuracy as compared to other classification algorithms. In a study conducted by Dawngliani et al. [17] to classify benign and malignant tumors for breast cancer screening dataset, J48 decision tree outperformed other decision tree algorithms in terms of the accuracy. The study tested J48 with other decision tree algorithms namely Decision Stump, Random Forest Tree, REP tree, Hoeffding Tree and Logistic Model Tree (LMT). In other study conducted by Mun and Jumadi [18], Naïve Bayes, Decision Table and Random Forest were tested to compare which algorithm produced the best statistical performance in classifying dyslexia dataset. The study has shown that Random Forest produced 100% accuracy and specificity in classifying dyslexia subject. Raj and Masood [19] classified ASD screening dataset by using Naïve Bayes, Support Vector Machine, Logical Regression, KNN, Neural Network, and Convolutional Neural Network (CNN). The study concluded CNN to be the best algorithm for handling classification as it has the highest accuracy as compared to other algorithms being tested.

The related works mentioned above showed that different classification techniques outperformed other classification techniques when tested to health related datasets. In relation to the mentioned related works, this study is intended to test the best classification technique from each existing study to the the ASD screening dataset in order to investigate which classification technique can produce better performance in screening the ASD class. We propose to investigate the performance of Naive Bayes, Logistic Regression, KNN, J48, Random Forest, and Deep Neural Network (DNN) algorithms by comparing their accuracy, specificity, sensitivity, receiver operating characteristic (ROC), and AUC results. We argue that Deep Neural Network (DNN) is most suitable to be used in replacement of CNN due to the fact that the ASD screening dataset contained no visual images or text embedding which does not fulfil the requirement for utilizing CNN. It is evident that CNN is mainly used to analyse and recognize patterns in images and text embeddings of a dataset by utilizing the input data’s collocated grids [20–22], thus, we replaced CNN with DNN instead.

Missing values are common in data analysis especially when big data from different sources is involved. Missing values or data inconsistency can potentially affect how data is analysed, and thus, it should be addressed by embedding several methods; deletion or data imputation [23–26]. Data imputation is a method whereby missing values are replaced with meaningful values such as the mean of the available records. This study aims at investigating the effect of ignoring missing values in the ASD dataset to the sensitivity, specificity, and accuracy of each classification algorithm used in this study since there exists a lack of studies being conducted to investigate this matter. Data imputation method has been utilized to address the missing values and to compare the sensitivity, specificity, and accuracy before and after the method was applied to the ASD dataset.

## Method

The main purpose of this study is to analyse a set of ASD screening data by using six classification algorithms to help in improving the diagnosis process of ASD in healthcare practices. This section demonstrates the approaches that we used to classify the ASD screening dataset including the classification process, classification techniques, performance matrix, and dataset involved in this study.

**Fig. 1:**
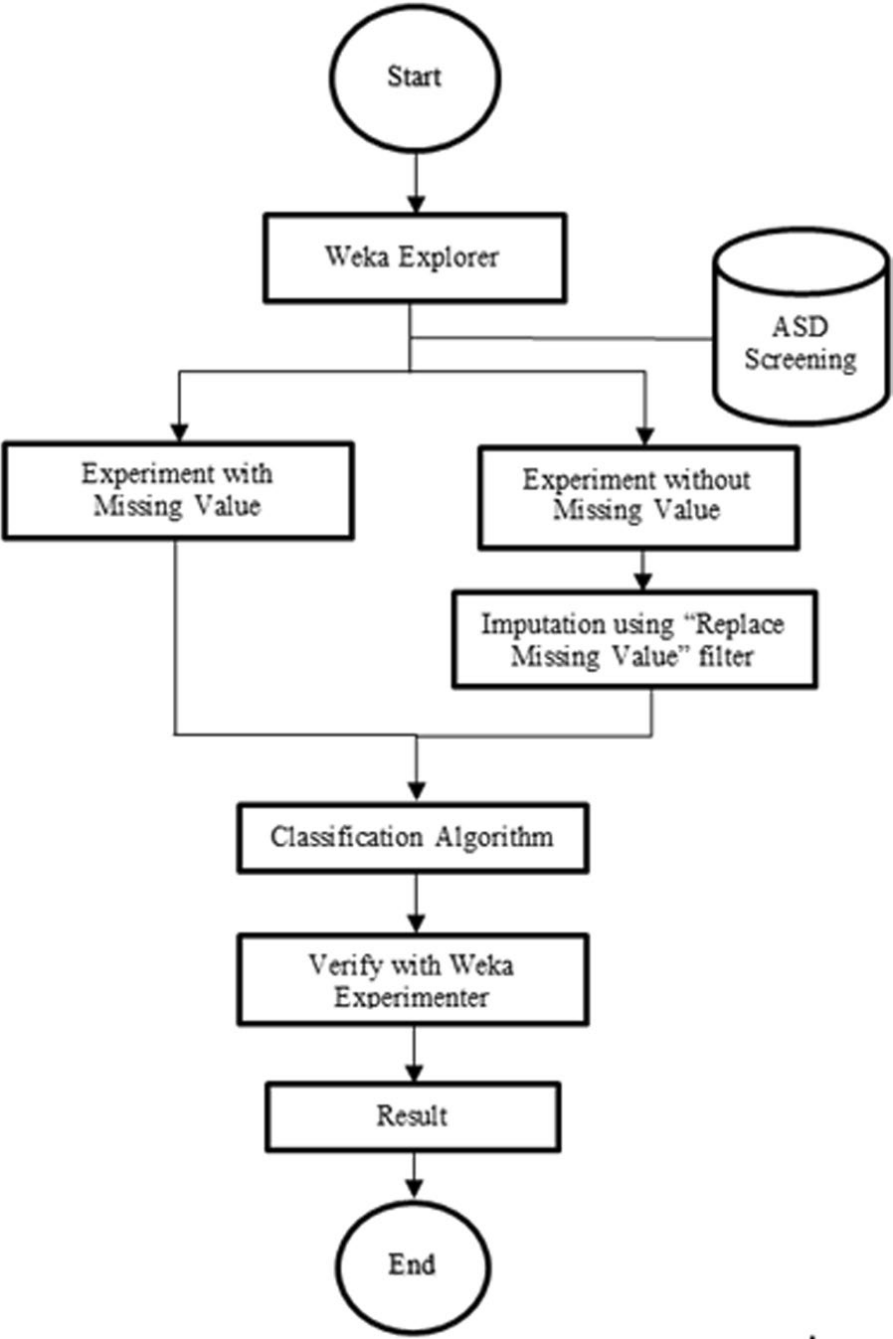
Classification process of ASD screening dataset

Since the output of the training dataset is known which is the screening class, classification technique is the most suitable one to be used in this case. Classication can help to improve screening process and assist at reducing any possible errors caused by inexperienced health practitioners [7, 27]. Figure 1 illustrates the process of classifying the dataset by using six chosen classifiers. The process began by classifying the ASD screening dataset in two conditions; with and without missing values, using six classifiers individually with Weka Explorer. As proposed by Tsai and Chang [28], data imputation method was used to deal with incomplete data by replacing the missing values with reasonable values. Once the results were obtained, we furthered the experiment by running all six classifiers simultaneously in Weka Experimenter and the results were analysed and compared for both conditions.

### Classification techniques

For the purpose of this study, six classifiers techniques have been chosen to analyse their performances in classifying ASD screening dataset. The classifiers used include Naive Bayes, Logistic Regression, KNN, J48, Random Forest, and DNN. Each of these classifiers have shown the highest performance when tested with other classifiers in previous studies.

Naïve Bayes uses Bayesian techniques to build a simple network in which it is assumed that the probability that one new example belongs to a class based on the assumption that all attributes are self-reliant from each other [29, 30]. According to Lewis [31], Naive Bayes algorithm can produce an optimal classifier in different situations where the assumptions are wildly violated. Logistic Regression is a multivariate statistical method which requires less assumption. The method is useful for evaluating the relationship between independent variables and dependent variables as well as for predicting the risk of a disease based on predictor variables built within the model [32].

K-nearest Network (KNN), or known as IBk in WEKA modeler environment, is an instance-based learning algorithm that is capable at classifying many types of datasets as well as capable of performing distance weighting. In KNN, when k samples belong to a type of category and it is found that the samples of the dataset are closely identical to other k samples, the samples will be classified into the category [33]. The classifier will examine the distance of the samples to the sample of the nearest neighbouring training in the feature space [34]. J48 is one of the decision tree techniques implemented from C4.5 algorithm. It consists of four main programmes; the decision tree generator, production rule generator, decision tree interpreter, and production rule interpreter. The decision tree produced by C4.5 is utilized for classification and grouping purposes. This algorithm works by producing a decision tree based on the dataset by recursive division of the data followed by the development of the decision tree using Depth-First strategy [35].

Random Forest, used for classifying and regressing an observation, is one of the decision tree techniques which is constructed by using a different bootstrap sample from the original data. The technique which roots from decision tree algorithm consists of a huge number of individual decision trees which work as an ensemble [36]. Approximately one-third of the data will be left out of the bootstrap sample and not utilized in the construction of the kth tree [37]. Similar to other classifiers that have been previously mentioned, Deep Neural Network (DNN) is one of the types of neutral network algorithms which can also be used for both classification and regression cases. It has been widely used as a tool to classify data in many crucial fields which can be extended from speech recognition to computational medicine [38]. The neural network incorporates the training of neural network with an amplified cross-entropy error function [39].

### Dataset

This study utilized a set of ASD screening dataset provided by Dr. Fadi Fayez Thabtah from the Department of Digital Technology, Manukau Institute of Technology, New Zealand 13]. The researcher developed a screening app used to screen the symptoms of ASD in patients and the data of the patients, which have been proven important in screening ASD occurrence 40], were collected and stored in a designated database.

The dataset is made up of 21 attributes, which includes 292 instances or records. The attributes or data fields include age, gender, ethnicity, jaundice status, existence of autism among family member, relation, country of residence, used screening app before, type of screening method, questions about the performance or ability of the patient (A1_Score, A2_Score, A3_Score, A4_Score, A5_Score, A6_Score, A7_Score, A8_Score, A9_Score, A10_Score), screening score, and screening class. The diagnosis result from the screening test is categorized into “1” for a patient diagnosed with ASD and “0” for a patient not diagnosed with ASD and due to the categorization, the dataset is categorized as asymmetric dataset. The dataset contains 90 or 30.82% missing values in the age, ethnicity, and relation attributes. Age is one of the most significant information needed in ASD screening as it assists practitioners to determine the type of early treatment that the patients should be undergoing if they are diagnosed with ASD [13, 41, 42]. Thus, it is important to address the missing value. Table 1 shows the list of the attribute names and types contained in the ASD screening dataset.

**Table 1:** Attributes of ASD screening dataset

Attributes name	Attribute code	Description	Attributes type
Age	Age	The child’s age	Number
Gender	Gender	The child’s gender (Male or Female)	String
Ethnicity	Ethnicity	List of common ethnicities in text format	String
Born with jaundice	Jaundice	Whether the child was born with jaundice	(0,1)
Family member with ASD	Autism	Whether any immediate family member has ASD	(0,1)
Relation	Relation	Parent, self, caregiver, medical staff, etc.	String
Country of residence	Country_of_res	Name of countries in text format	String
Used the app before	Used_app_before	Whether the user has used a screening app	Boolean (yes or no)
Screening Method Type	Age_desc	The type of screening methods chosen based on age category (0 = toddler, 1 = child, 2 = adolescent, 3 = adult)	Integer (0,1,2,3)
Question 1	A1_Score	Question asking “Does your child look at you when you call his or her name?”	(0,1)
Question 2	A2_Score	“How easy is it for you to get eye contact with your child?”	(0,1)
Question 3	A3_Score	“Does your child point to something to indicate that he or she wants something?”	(0,1)
Question 4	A4_Score	Does your child point to something to share interest with you?	(0,1)
Question 5	A5_Score	Does your child pretend? (e.g.: talk on the toy phone)	(0,1)
Question 6	A6_Score	Does your child follow where you are looking at?	(0,1)
Question 7	A7_Score	If you or someone else in the family is visibly upset, does your child show sign of wanting to comfort them?	(0,1)
Question 8	A8_Score	How would you describe your child’s first word?	(0,1)
Question 9	A9_Score	Does your child use simple gesture? (example: waving for goodbye)	(0,1)
Question 10	A10_Score	Does your child stare at nothing with no apparent purpose?	(0,1)
Screening Score	Result	The final score obtained based on the scoring algorithm of the screening method used. This was computed in an automated manner	Integer
Screening Class	Class/ASD	Whether the child has ASD or not	Boolean (yes or no)

**Fig. 2:**
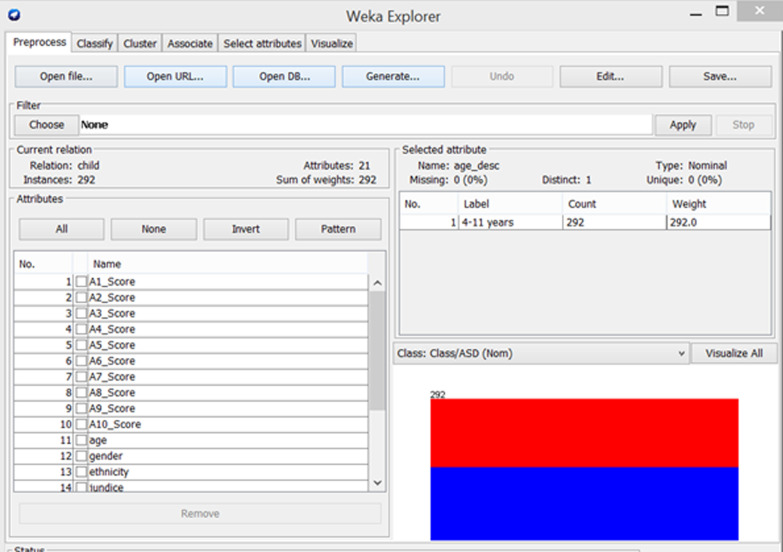
Description of ASD screening dataset in WEKA

When the ASD screening dataset is loaded to WEKA modeler environment, the modeler will recognize the attributes and during the scanning process of the dataset, it will compute descriptive statistical analysis (Table 2) on each attribute in the dataset. The left panel highlighted in green (Fig. 2) shows the list of attributes for the dataset. The modeler shows that the dataset contains 21 attributes and 292 instances in total.

**Table 2:** Descriptive statistical analysis of ASD screening dataset before normalization

Attribute code	Attribute value	N	Min.	Max.	Mean	SD
Age	4–11	288	4	11	6.354	2.365
Gender	F	84	n/a	n/a	n/a	n/a
	M	208	n/a	n/a	n/a	n/a
Ethnicity	Asian	46	n/a	n/a	n/a	n/a
	Black	14	n/a	n/a	n/a	n/a
	Hispanic	7	n/a	n/a	n/a	n/a
	Latino	8	n/a	n/a	n/a	n/a
	Mid- Eastern	27	n/a	n/a	n/a	n/a
	Pasifika	2	n/a	n/a	n/a	n/a
	South Asian	21	n/a	n/a	n/a	n/a
	Turkish	2	n/a	n/a	n/a	n/a
	White-European	108	n/a	n/a	n/a	n/a
	Others	14	n/a	n/a	n/a	n/a
Jaundice	No	212	n/a	n/a	n/a	n/a
	Yes	80	n/a	n/a	n/a	n/a
Autism	No	243	n/a	n/a	n/a	n/a
	Yes	49	n/a	n/a	n/a	n/a
Relation	Parent	214	n/a	n/a	n/a	n/a
	Self	5	n/a	n/a	n/a	n/a
	Relative	17	n/a	n/a	n/a	n/a
	Healthcare professional	13	n/a	n/a	n/a	n/a
Used_app_before	No	281	n/a	n/a	n/a	n/a
	Yes	11	n/a	n/a	n/a	n/a
A1_Score	0	107	n/a	n/a	n/a	n/a
	1	185	n/a	n/a	n/a	n/a
A2_Score	0	136	n/a	n/a	n/a	n/a
	1	156	n/a	n/a	n/a	n/a
A3_Score	0	75	n/a	n/a	n/a	n/a
	1	217	n/a	n/a	n/a	n/a
A4_Score	0	131	n/a	n/a	n/a	n/a
	1	161	n/a	n/a	n/a	n/a
A5_Score	0	75	n/a	n/a	n/a	n/a
	1	217	n/a	n/a	n/a	n/a
A6_Score	0	84	n/a	n/a	n/a	n/a
	1	208	n/a	n/a	n/a	n/a
A7_Score	0	115	n/a	n/a	n/a	n/a
	1	177	n/a	n/a	n/a	n/a
A8_Score	0	147	n/a	n/a	n/a	n/a
	1	145	n/a	n/a	n/a	n/a
A9_Score	0	148	n/a	n/a	n/a	n/a
	1	144	n/a	n/a	n/a	n/a
A10_Score	0	80	n/a	n/a	n/a	n/a
	1	212	n/a	n/a	n/a	n/a
Result	n/a	292	0	10	6.24	2.285
Class/ASD	No	151	n/a	n/a	n/a	n/a
	Yes	141	n/a	n/a	n/a	n/a

### Performance metrics

In this study, the accuracy result of each classifier is used as the performance metrics for the purpose of analysing each classifier’s performance more systematically. The AUC metric is also examined for all classifiers used in this study. The metric is used to measure the performance of each classifier over the full range of sensitivities and specificities and is not affected by the trade-off between accuracy and specificity [30].

#### Confusion matrix

Confusion matrix is used to evaluate the performance of the classification algorithms. It is a visualization tool used to illustrate the accuracy, specificity, and sensitivity of the classifiers in classification process. The matrix illustrates the relationships between the outcome and prediction of classes shown in Table 3.

**Table 3:** Confusion matrix

	Predicted	
Actual	Positive	Negative
Positive	a	b
Negative	c	d

The description of the confusion matrix shown in Table 2 is shown as follows:

- a is True Positive (TP) where it is predicted as having ASD and having ASD in actual situation.
- b is False Negative (FN) where it is predicted as not having ASD but having ASD in actual situation.
- c is False Positive (FP) where it is predicted as having ASD but not having ASD in actual situation.
- d is True Negative (TN) where it is predicted as not having ASD and not having ASD in actual situation.

In data mining, accuracy, calculated using the formula; (TP + TN)/(TP + FP + TN + FN), is defined as the proportion of the total number of data that is correctly classified. It is computed by dividing the total sum true positive and true negative with the total sum of all positive and negative data. Sensitivity or recall or true positive rate, calculated using the formula; TP/(TP + FN), calculates the proportion of actual positives that are correctly classified, for instance, the percentage of patients that are correctly identified as having ASD. To calculate accuracy from the confusion matrix, the total number of data categorized as true positive is divided with the total sum of true positive and false negative data. Specificity or true negative rate calculates the proportion of actual negatives that are correctly classified, for instance, the percentage of patients correctly identified as not having ASD. The formula used is TN/(TN + FP). Sensitivity is calculated from true negative and false positive data. Specificity also allows practitioners to determine the probability of false alarm.

#### ROC and AUC

The performance of each algorithm can also be measured by comparing the result of ROC and AUC. ROC plots the classification algorithm’s sensitivity and specificity at different classification thresholds in a visual format. Meanwhile, AUC measures the area below the ROC curve which is depicted as the probability that the classification algorithm ranks a random positive data more highly than a random negative data.

### Pre-processing and processing measures

The ASD screening dataset contains missing values in three attributes; age, ethnicity, and relation, thus the study tested the classifiers on the dataset with and without missing values to investigate the effect of both conditions on the classifiers’ performance result. The missing values were addressed during the pre-processing phase by deploying the most used imputation method which is imputation using mean values. Imputation using mean values is relevant in this study since the missing values in the ASD screening dataset are categorized as missing completely at random (MCAR), thus reducing the biasness to the classification result [43]. Moreover, the imputation method is performed to the dataset as it is easy and less time consuming as well it is suitable for small dataset such as the ASD screening dataset used in this study. We also performed normalization technique to numeric attributes namely age and result by using “Normalize” function on WEKA Explorer.

After the pre-processing stage, cross-validation mode was performed to the dataset by splitting the data into k-block or a chunk of objects and this process was performed before classification process took place. Once the modeler split the dataset, the data was tested by each classifier in which it was trained to use K-1 block and the process was reiterated for all the blocks. For this study, the ASD screening dataset was split into ten blocks.

## Results and discussion

This section presents the results of the classification process when we tested the classifiers using WEKA Explorer and WEKA Experimenter to the dataset in two conditions; with and without missing values by which the results are presented and discussed accordingly.

### Testing classifier performance using WEKA explorer

**Fig. 3:**
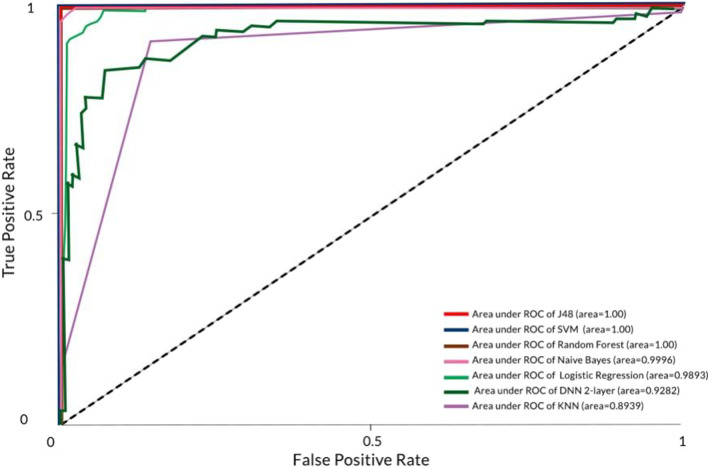
AUC of classifiers for ASD ‘yes’ class (with missing values)

The study tested six different types of classifiers which include Naïve Bayes, Logistic Regression, KNN, J48, Random Forest, DNN, and SVM. As for DNN classifier, we utilized two-layer technique. Table 4 illustrates the performance of the classifiers tested in WEKA Explorer by deploying cross-validation test mode. For classification with missing values, the area under ROC curve (AUC) of each classifier has also been illustrated for comparison as shown in Fig. 3.

**Table 4:** Weighted average of classifiers for ASD dataset with missing values

Classifier	Training runtime (s)	Correctly classified instance % (Accuracy)	Incorrectly classified instance %	Kappa statistic	TP rate	FP rate	Precision (sensitivity)	Recall (specificity)	ROC	AUC
Naïve Bayes	0.03	98.9726	1.0274	0.9794	0.990	0.010	0.990	0.990	1.000	0.9996
Logistic regression	0.51	95.2055	4.7945	0.904	0.952	0.048	0.952	0.952	0.991	0.9893
KNN	0.00	88.3562	11.6438	0.7674	0.884	0.114	0.886	0.884	0.894	0.8939
J48	0.08	100.0000	0.0000	1.000	1.000	0.000	1.000	1.000	1.000	1.000
Random Forest	0.76	100.0000	0.0000	1.000	1.000	0.000	1.000	1.000	1.000	1.000
DNN (2-layer)	28.93	86.9863	13.0137	0.7393	0.870	0.131	0.870	0.870	0.928	0.9282
SVM	0.88	100.0000	0.0000	1.000	1.000	0.000	1.000	1.000	1.000	1.000

**Fig. 4:**
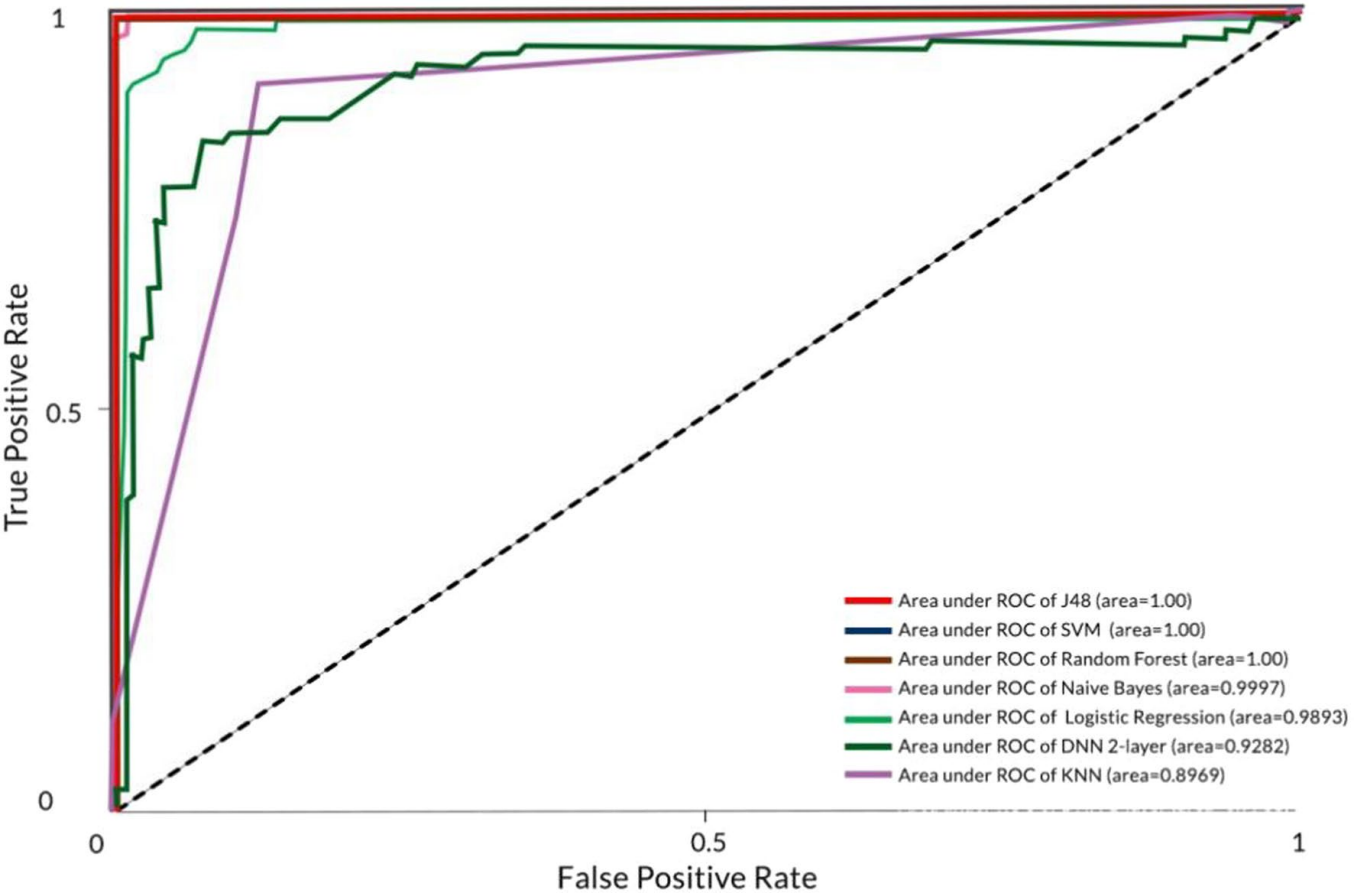
AUC of each classifier for ASD ‘yes’ class (without missing values)

We furthered the study by classifying the ASD screening dataset through the removal of the missing values in the dataset. This was conducted by using one of the imputation methods which is imputation using mean values. In WEKA modeler environment, the said imputation method is represented as “Replace Missing Value” filter. In the pre-processing phase, the missing values were replaced with the mean value of the attribute that contained missing values, before being classified using the classifiers. The result is organized in Table 5. We also illustrated the AUC of each classifier for comparison in Fig. 4.

**Table 5:** Weighted average of classifiers for ASD dataset without missing values

Classifier	Training runtime (s)	Correctly classified instance % (Accuracy)	Incorrectly classified instance %	Kappa statistic	TP rate	FP rate	Precision (sensitivity)	Recall (specificity)	ROC	AUC
Naïve Bayes	0.03	98.9726	1.0274	0.9794	0.990	0.010	0.990	0.990	1.000	0.9997
Logistic regression	0.11	95.2055	4.7945	0.9040	0.952	0.048	0.952	0.952	0.991	0.9893
KNN	0.00	89.3836	10.6164	0.7878	0.894	0.105	0.895	0.894	0.897	0.8969
J48	0.01	100.0000	0.0000	1.0000	1.000	0.000	1.000	1.000	1.000	1.000
Random Forest	0.27	100.0000	0.0000	1.0000	1.000	0.000	1.000	1.000	1.000	1.000
DNN (2-layer)	26.29	86.9863	13.0137	0.7393	0.870	0.131	0.870	0.870	0.928	0.9282
SVM	0.80	100.0000	0.0000	1.000	1.000	0.000	1.000	1.000	1.000	1.000

From the experiments, all classifiers obtained different results when classifying the ASD screening dataset with and without missing values. Naïve Bayes classifier produced slightly different AUC score when classifying the dataset with and without imputation, dismissing the previous studies [44, 45] which claimed that Naïve Bayes is insensitive to missing values. Based on the classification result of all the classifiers being tested in Table 4, when missing values were ignored, J48, Random Forest, and SVM have shown 100% performance as compared to other classifiers in terms of the accuracy, sensitivity, and specificity. However, J48 only required 0.08 s to classify the dataset as compared to Random Forest (0.76 s) and SVM (0.88 s). Meanwhile, DNN produced the lowest accuracy result (86.9863%) in classifying the dataset with missing values in comparison to KNN (88.3562%), Logistic Regression (95.2055), and Naïve Bayes (98.9726%). In terms of the computational complexity to build the classifying model, DNN has shown the worst performance in which it required 28.93 s to build the model followed by Random Forest (0.76 s), Logistic Regression (0.51 s), SVM (0.88 s), J48 (0.08 s), and Naïve Bayes (0.03 s). Despite the fastest run time (0.00 s) in classifying the dataset, KNN failed to classify 11.6438% of instances correctly when missing value were ignored, the second highest after DNN.

Based on Table 5, when the missing values were replaced with the mean values, both J48 and Random Forest classifiers have shown constant performance similar to when missing values were ignored except for the time taken to build the model which has increased for both classifiers; 0.01 s for J48 and 0.27 s for Random Forest. The time taken for all classifiers to build the model has also improved with KNN producing the fastest speed to build the model (0.00 s) followed by J48 (0.01 s), Naïve Bayes (0.03 s), Logistic Regression (0.11 s), Random Forest (0.27 s), SVM (0.80 s), and DNN (26.29 s). However, KNN failed to classify 10.6164% of instances correctly as compared to other classifiers that required more time to classify the instances but can produce better accuracy.

Further, we compared the ROC score to study the performance of each classifier. ROC enables the demonstration of the diagnosis ability of a classifier in which the main goal is to have the curve closer to the value 1 on the Y-axis of the graph which indicates the ability of a classifier to classify a given dataset accurately. Simply put, the closer the curve to the value 1, the better the performance of the classifier at classifying a dataset. J48, Naïve Bayes, Random Forest, and SVM produced the best ROC curve which indicates that the classifiers can predict ASD occurrences accurately as opposed to Logistic Regression, KNN, and DNN when classifying the dataset in both conditions.

From the ROC curve, we further illustrated the AUC of each classifier shown in Figs. 3 and 4. The experiment showed that when the dataset with missing values is classified, J48, SVM, and Random Forest produced the best AUC with 1.000 score followed by Naïve Bayes (0.9996), Logistic Regression (0.9893), DNN (0.9282), and KNN (0.8939). When testing the dataset without the missing values, Naïve Bayes and KNN increased their AUC performances by 0.0001 from 0.9996 to 0.9997 and by 0.03 from 0.839 to 0.869 respectively. The result indicates that the classifiers that could not produce a perfect score of 1.000 for AUC are unable to predict ASD occurrences accurately, this in turn, may affect the life of a patient being wrongly diagnosed.

### Verifying classifier performance using WEKA experimenter

We further the experiment by classifying the dataset with and without missing values by utilizing WEKA Experimenter in order to validate the results produced by the classifiers when tested in WEKA Explorer environment. WEKA Experimenter allows more than one classifier to be tested simultaneously to classify the dataset. We tested the dataset by running tenfold or 10-epoch cross-validation test mode.

Corrected Paired T-Test mode was utilized to verify the performance of each classifier by comparing the accuracy and AUC results of each of the classifiers when tested simultaneously. The test works by comparing each classifier in pairs and making reasonable assumptions with regards to the distribution of the results collected. The test used 0.05 two-tailed confidence level and the result is shown in Table 6.

**Table 6:** Accuracy and AUC score of all classifiers tested simultaneously in WEKA Experimenter (with missing values)

Classifier	Correctly classified instance % (accuracy)	AUC
Naïve Bayes	98.84	1.00
Logistic Regression	95.51	0.99
KNN	88.42	0.89
J48	100.00	1.00
Random Forest	99.97	1.00
DNN (2-layer)	94.52	0.98
SVM	100.00	1.00

Based on the test conducted using WEKA Experimenter, 600 data were loaded since each classifier was evaluated 100 times (tenfold cross validation multiplied by 10 repetitions). We compared the accuracy and AUC score of each classifier when tested in both WEKA Explorer and WEKA Experimenter. The result shows that J48 and SVM classifier outperformed other classifiers in terms of its accuracy and AUC readings; 100% and 1.00 respectively. On the other hand, the accuracy of Naïve Bayes and Random Forest decreased while Logistic Regression, KNN, and DNN improved their accuracy especially DNN that produced 7.5337% of improvement. We furthered the test by using J48 as the test base since J48 has better training runtime than SVM.The result shows that no classifiers outperformed the test base, except Random Forest, which generated 1.00 of AUC result, akin to J48.

In addition, the accuracy of Logistic Regression (95.51%), DNN (94.52), and KNN (88.42%) as well as the AUC of KNN (0.89) and DNN (0.98) have shown to be significantly different than that of J48.

Based on the test using both WEKA Explorer and WEKA Experimenter, it is evident that J48 is the best classifier among the other five classifiers with 95% of confidence level when tested with the dataset containing missing values. This shows that J48 is capable at handling dataset that contains missing values. Further, a study conducted by Aziz et al. [46] tested Naïve Bayes, J48, and Random Forest to classify a non-medical dataset and the classification results were compared. Based on the result, they found that Naïve Bayes produced the best accuracy in classifying the instances as opposed to this study. Thus, we can suggest that a classifier’s classifying performance is affected by the type of dataset as well as the number of instances involved in the experiment.

We then addressed the missing values by using imputation method and tested the dataset in WEKA Experimenter. We tested the dataset without missing values by deploying the same method through which we used Corrected Paired T-Test by using 0.05 significance level and compared the performance of each classifier in terms of the accuracy and AUC score. Since J48 turned out to be the best classifier among other classifiers being tested, we used the classifier as the test base for the experiment. The result of the accuracy and AUC of the classifiers is illustrated in Table 7.

**Table 7:** Accuracy and AUC score of all classifiers tested simultaneously in WEKA Experimenter (without missing values)

Classifier	Correctly classified instance % (accuracy)	AUC
Naïve Bayes	98.91	1.00
Logistic regression	95.51	0.99
KNN	89.08	0.90
J48	100	1.00
Random Forest	100	1.00
DNN (2-layer)	94.52	0.98
SVM	100.00	1.00

As shown in Table 6, we found that Random Forest and SVM, both of which have 100% accuracy and 1.00 AUC score, have outperformed other classifiers when missing values were replaced with the mean values of the attributes containing missing values. The experiment has illustrated that Logistic Regression, KNN, and DNN to be significantly different than that of the test base in terms of the accuracy. Meanwhile, in terms of the result of AUC, KNN and DNN have shown to be significantly different than the test base. By comparing the classification result of ASD screening dataset in both conditions, the study shows that Random Forest can perform better when the dataset contains no missing values since the classifier has shown an improvement from 99.97% (“with missing values” condition) to 100.00% (“without missing values” condition) of reading when classifying the ASD class correctly. This is due to its nature of being capable at providing the best estimates of the variables that are deemed important in the classification. Besides, the classifier is also capable at estimating the missing values and maintaining its accuracy when the amount of missing values is massive. On the other hand, Logistic Regression and DNN are not affected by the existence of missing values in the dataset. We also found that Naive Bayes improved its accuracy when missing values were replaced with the mean value of the attribute. This study has somewhat debunked the study which showed that Naïve Bayes has low sensitivity towards the existence of missing values in a dataset [47]. However, a further study is sought to test its sensitivity using other datasets.

#### Confusion matrix of the best classifier

**Fig. 5:**
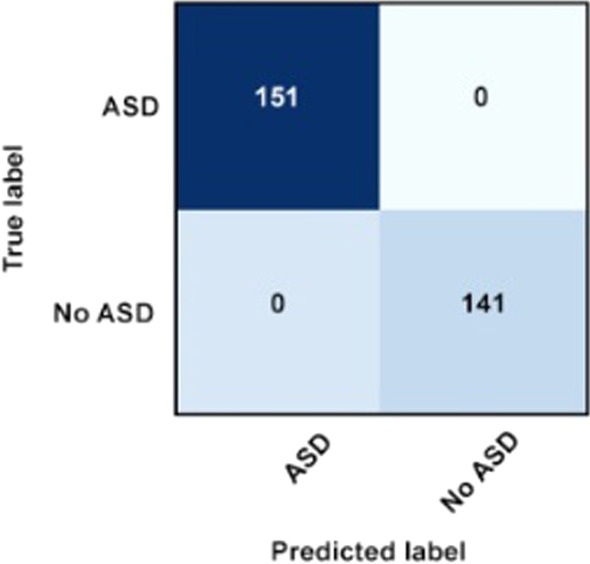
Confusion Matrix of J48 for ASD screening dataset with missing values

**Fig. 6:**
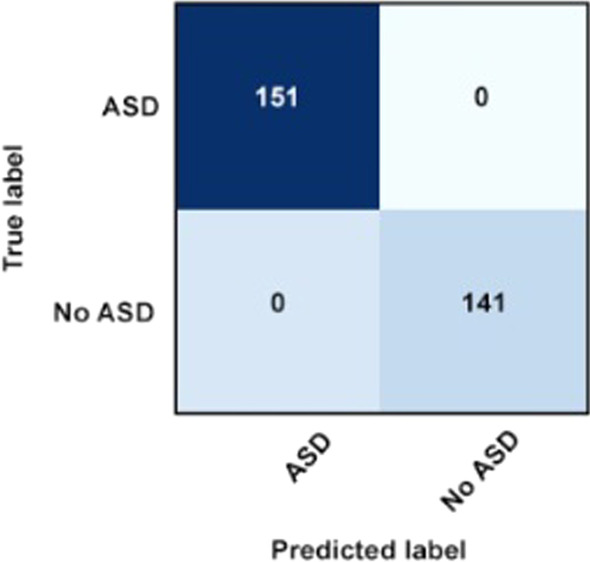
Confusion Matrix of J48 for ASD screening dataset without missing values

As mentioned previously, confusion matrix is a visualization tool that allows researchers to illustrate the accuracy, specificity, and sensitivity of a given classifier. From the experiment, confusion matrix is produced to illustrate the accuracy, specificity, and sensitivity of J48 classifier, the best classifier among other classifiers being tested. The confusion matrix of J48 classifier is shown in Figs. 5 and 6 for both conditions; ASD screening dataset with and without missing values, respectively.

The confusion matrix which is produced when J48 is used to classify ASD screening dataset with and without missing values illustrates that in both conditions, J48 could classify 151 instances with ‘yes’ class category and 141 instances with ‘no’ class category correctly. The matrix also shows that neither false positive nor false negative classification was produced by J48, which proves that J48 can perform better in classifying the database in both conditions as compared to other classifiers especially Random Forest which also produced the same accuracy score as J48 in WEKA Explorer but can only produce 99.97% of accuracy when tested in WEKA Experimenter environment when missing values were present in the ASD dataset.

## Future work and conclusion

Not many studies have utilized classification algorithms to particularly analyse ASD screening dataset. From the literature review, most researchers utilized other most common datasets such as breast cancer and heart disease datasets, thus there exists difficulties in analysing and comparing the performance of classifiers being tested to ASD screening dataset. This is important in order to assist in selecting the best classification methods for screening and diagnosing a patient with ASD, thus we attempted to fill in the gap by analysing and classifying ASD screening dataset by using six classifiers. Based on the result obtained through the tests with both Weka Explorer and Weka Experimenter using tenfold cross validation, we can conclude that J48 outperformed other classifiers in terms of the time taken to classify the class as well as the accuracy and AUC produced by the classifier when tested to the ASD dataset with and without missing values. On the other hand, KNN performed poorly in classifying the dataset as compared to other classifiers even though the classifier took 0.00 s of runtime to classify the dataset.

The accuracy produced by J48 may assist health practitioners to make better decision when analysing datasets in both circumstances, with and without missing values. Making a right decision at the right time is imperative in crucial industries especially health-related field for it to perform effectively and efficiently as well as to reduce human errors which can affect the life of a patient. Moreover, we compared our results with the previous works mentioned in this paper and found that a classifier’s classifying performance is affected by the type of dataset as well as the number of instances involved in the experiment. However, a specific study is required to test the classification algorithms to classify other health related datasets with different volume of instances in order to investigate the effect of the volume on the performance of the algorithms. Future work can include utilizing other classification algorithms such as Simple Cart, Ada Boost, Logit Boost, Bagging, and Decision Stump. This study can also be further extended by deploying multi-modal deep learning to predict ASD occurrences as well as the abnormalities of other related neurological diseases such as amnestic mild cognitive impairment and multiple sclerosis [48, 49]. Simulating missing values existing in other significant attribute fields should also be considered for future work. Moreover, other imputation methods are sought to be used for addressing the missing values as the current imputation method used in this study may lead to inaccuracy and uncertainty when testing the dataset. Lastly, an extensive study is suggested to be carried out to further investigate the kappa value of each classifer being tested in this study.

## Data Availability

The datasets used in this study is a public dataset provided by Dr. Fadi Fayez Thabtah from the Department of Digital Technology, Manukau Institute of Technology, New Zealand. The dataset is available publicly at this website: https://archive.ics.uci.edu/ml/datasets/Autism+Screening+Adult.
